# Supplementary material for: Liver Expression of IGF2 and Related Proteins in ZBED6 Gene-Edited Pig by RNA-Seq
Source: Animals (Basel). 2020 Nov 22;10(11):2184. doi: 10.3390/ani10112184 (PMC7700129; doi:10.3390/ani10112184)
Supplement: Supplementary file 1 [file animals-10-02184-s001.pdf]

Table S1 Blood physiological index of *ZBED6* KO pig and wild type.

blood physiological index	KO (mean±SD)	SKO (mean±SD)	Wild type (mean±SD)	*P* value	Unit
WBC	18.37±1.17	17.87±1.74	18.65±2.97	0.901	10^9/L
NEU#	6.38±1.64	5.12±0.26	7.32±0.81	0.113	10^9/L
LYMPH#	9.75±1.85	10.33±1.25	9.79±3.62	0.949	10^9/L
MONO#	1.12±0.07[a]	1.03±0.06[ab]	0.82±0.20[b]	0.070	10^9/L
EO#	0.77±0.24	1.16±0.76	0.52±0.38	0.361	10^9/L
BASO#	0.36±0.13	0.23±0.03	0.19±0.13	0.213	10^9/L
NEU%	34.87±9.12	28.87±3.5	40.37±10.45	0.305	%
LYMPH%	53.00±8.36	57.77±3.78	51.40±11.32	0.649	%
MONO%	6.07±0.42[a]	5.77±0.29[ab]	4.33±0.47[b]	0.004	%
EOS%	4.17±1.08	6.33±3.49	2.83±1.96	0.272	%
BASO%	1.90±0.60	1.27±0.12	1.07±0.70	0.221	%
RBC	7.91±0.19	7.61±0.48	7.68±1.12	0.865	10^12/L
HGB	155.33±13.01	144.67±4.04	162.67±24.38	0.44	g/L
HCT	49.07±3.56	43.90±0.79	49.83±8.02	0.362	%
MCV	62.00±4.00	57.87±3.58	64.83±2.55	0.117	fL
MCH	19.63±1.50	19.10±1.30	21.20±0.50	0.157	Pg
MCHC	316.33±4.16[b]	330±3.61[a]	326.67±5.86[ab]	0.026	g/L
RDW-CV	19.50±1.73	18.07±1.27	17.70±1.47	0.364	%
RDW-SD	41.33±1.53	36.33±1.15	39.33±4.04	0.135	L
PLT	219.67±90.91	209.33±81.99	321.67±55.29	0.226	10^9/L
MPV	9.77±2.11	9.23±0.71	9.53±0.91	0.897	fL
PDW	14.90±0.10	14.97±0.21	15.20±0.26	0.242	fL
PCT	0.22±0.11	0.19±0.07	0.31±0.08	0.311	%
P-LCC	71.33±57.07	52.67±17.67	89.67±38.84	0.576	10^9/L
P-LCR	31.03±17.75	26.03±6.34	27.13±7.56	0.863	%

Notes: KO: knockout; SKO: single knockout; WBC: White blood cell count; NEU#: neutrophil number; LYMPH#: lymphocyte number; MONO#: monocyte number; EO#: eosinophil number; BASO#: basophil number; NEU%: neutrophil percentage; LYMPH%: lymphocyte percentage; MONO%: monocyte percentage; EOS%: eosinophil percentage; BASO%: basophil percentage; RBC:

Red blood cell number; HGB: hemoglobin; HCT:hematocrit; MCV: average red blood cell volume; MCH: average red blood cell hemoglobin amount; MCHC: average red blood cell hemoglobin concentrated; RDW-CV: red blood cell distribution width variation; RDW-SD: red blood cell distribution width standard deviation; PLT: platelet number; MPV: average platelet volume; PDW: platelet distribution width, PCT: small blood; P-LCC: large platelet count; P-LCR: large platelet ratio.

Table S2 Serum biochemical index of *ZBED6* KO pig and wild type.

**serum biochemical index**	**KO (mean±SD)**	**SKO (mean±SD)**	**Wild type (mean±SD)**	***P* value**	**Unit**
TP	71.67±6.88	68.53±3.72	70.4±6.87	0.819	g/L
Alb	39.87±6.38	35.47±3.57	36.67±4.24	0.555	g/L
ALT	44.67±2.52	43±18.03	41±15.72	0.949	U/L
AST	25.67±5.77	30±9.54	30.67±5.86	0.676	U/L
ALP	65.67±18.45	84.33±61.45	193.33±138.35	0.238	U/L
Urea	4.86±0.37	3.81±0.86	3.58±0.72	0.122	mmol/L
TG	0.55±0.14[b]	1.04±0.36[ab]	1.3±0.44[a]	0.086	mmol/L
HDL-C	1.17±0.33	0.97±0.13	0.88±0.19	0.337	mmol/L
LDL-C	1.04±0.16	1.04±0.14	0.93±0.15	0.600	mmol/L
LDH	396±103.06	422.67±110.53	333±7.55	0.479	IU/L

Notes：KO：knockout; SKO：single knockout; TP: Total protein; Alb: albumin; ALT: alanine aminotransferase; AST: aspartate aminotransferase; ALP: alkaline phosphatase; TG: triglyceride; HDL-C: high density lipoprotein cholesterol; LDL-C: low density lipoprotein cholesterol; LDH: lactate deaerase.

Table S3 GO and KEGG pathways with significant *P* value

Category	Term	Count	Genes	*P* Value
KEGG_PATHWAY	ssc00061:Fatty acid biosynthesis	2	ACACA, ACSBG1	0.049032894
KEGG_PATHWAY	ssc04668:TNF signaling pathway	3	TNFRSF1B, EDN1, RIPK3	0.064455591
GOTERM_MF_DIRECT	GO:0005524~ATP binding	10	ACTG1, GSS, RECQL, ATP2B4, MYO7A, DDX39B, ACACA, RIPK3, PKN2, CDK10	0.010846371
GOTERM_CC_DIRECT	GO:0000346~transcription export complex	2	DDX39B, NXF1	0.014571141
GOTERM_BP_DIRECT	GO:0033077~T cell differentiation in thymus	2	ZFP36L1, RIPK3	0.063257527
GOTERM_BP_DIRECT	GO:0034097~response to cytokine	2	RELB, ACP5	0.06968484
GOTERM_BP_DIRECT	GO:0007249~I-kappaB kinase/NF-kappaB signaling	2	RELB, RIPK3	0.079245316
GOTERM_BP_DIRECT	GO:0035914~skeletal muscle cell differentiation	2	MEF2D, BCL9	0.085565711
GOTERM_BP_DIRECT	GO:0000122~negative regulation of transcription from RNA polymerase II promoter	4	PHF14, RELB, EDN1, NR0B2	0.092808295

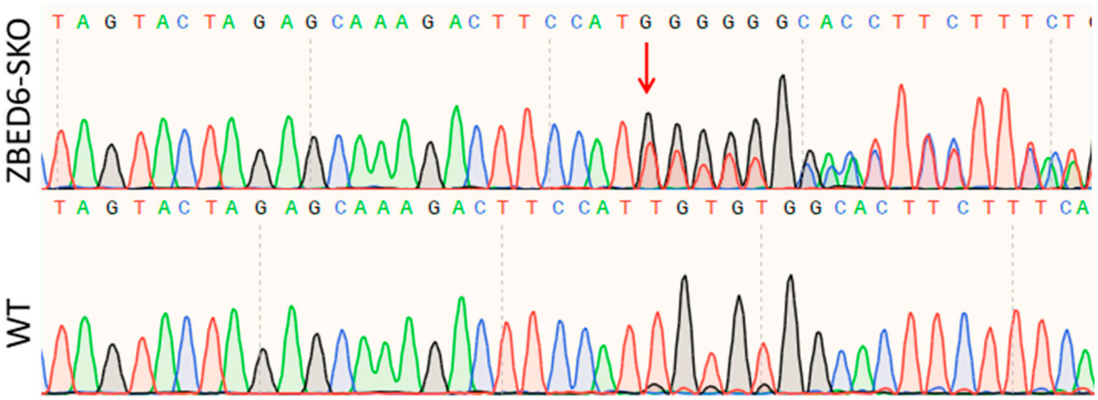

Figure S1 sequencing of *ZBED6*-KO locus and *IGF2* 3072 locus
